# Pre-existing comorbidity modify emergency room visit for out-of-hospital cardiac arrest in association with ambient environments

**DOI:** 10.1371/journal.pone.0204593

**Published:** 2018-09-26

**Authors:** Yu-Chun Wang, Yi-Chun Chen, Chun-Yu Ko, Yue-Liang Leon Guo, Fung-Chang Sung

**Affiliations:** 1 Department of Environmental Engineering, College of Engineering, Zhongli, Taiwan; 2 Research Center for Environmental Risk Management, Chung Yuan Christian University, Zhongli, Taiwan; 3 Department of Health Management, I-Shou University, Kaohsiung, Taiwan; 4 Department of Environmental and Occupational Medicine, National Taiwan University College of Medicine, Taipei, Taiwan; 5 Division of Environmental Health and Occupational Medicine, National Health Research Institutes, Zhunan, Miaoli County, Taiwan; 6 Department of Health Services Administration, China Medical University, Taichung, Taiwan; 7 Management Office for Health Data, China Medical University Hospital, Taichung, Taiwan; Azienda Ospedaliero Universitaria Careggi, ITALY

## Abstract

**Background:**

This study evaluated risks of emergency room visit (ERV) for out-of-hospital cardiac arrest (OHCA) in 2005–2011, among patients with cardiologic and metabolic syndromes (CMS), in association with ambient environments.

**Methods:**

Pooled and area-specific weather related cumulative six-day (lags 0 to 5) relative risks (RRs) and confidence intervals (CIs) of ERV for OHCA were evaluated for CMS cases, using distributed lag nonlinear models and multivariate meta-analytical second-stage model in association with the daily average temperatures and daily concentrations of air pollutants.

**Results:**

ERV risk increased as average temperature dropped to <27°C. At the mean temperature of 14°C, the cumulative six-day RRs of ERV were 1.73 (95% CI: 1.22, 2.46) for all OHCA patients, 1.74 (95% CI: 1.06, 2.84) for OHCA patients younger than 65 years old, and 1.99 (95% CI: 1.03, 3.81) for subjects with pre-existing hypertension. High temperature was also associated with elevated ERV of OHCA. Increased ERV risks in cases with pre-existing hypertension and diabetes mellitus were also associated with concentrations of air pollutants in northern Taiwan.

**Conclusions:**

Our data provided evidences to clinicians, emerging medical services and public health that the ERV risk for OHCA patients is greater at low temperature than at high temperature. Patients with cardio and metabolic disorders need to pay greater attention to low temperature and avoid heat wave.

## Introduction

Sudden cardiac arrest is one of leading causes of deaths worldwide. Over 320,000 victims experienced out-of-hospital cardiac arrests (OHCAs) in the United States in 2011[1]. Older age and males, and comorbidities, such as diabetes mellitus, cardiovascular disorders, and hypertension, have been linked to an elevated OHCA risk[2–6].

Studies have correlated the incident OHCA to the ambient temperature[7–11]. The OHCA incidence is higher in cold season than when it is hot. However, significant elevation of OHCA during heat wave has been reported as well[9, 11].

Recent studies have also linked the risk of OHCA to ambient concentrations of air pollutants[10, 12–24] independent from ambient temperature[7, 11]. Particulate matter less than 10 or 2.5μm in aerodynamic diameter (PM_10_ or PM_2.5_)[10, 16, 20, 22–25], and ozone (O_3_)[12, 13, 21, 22, 24] have been identified as air pollutants associated with OHCA. But, the associations between OHCA and other gaseous air pollutants are inconsistent [12].

So far, no study has evaluated whether individuals with existing disorders living in subtropical and tropical areas are at the OHCA risk. Taiwan is an island located on the western part of the Pacific Ocean with subtropical climate[11, 23]. This study aims to evaluate emergency room visits (ERVs) for OHCA patients in association with ambient environment and health status, using claims data of a national representative population of one million people.

## Materials and methods

### Data sources

**Fig 1:**
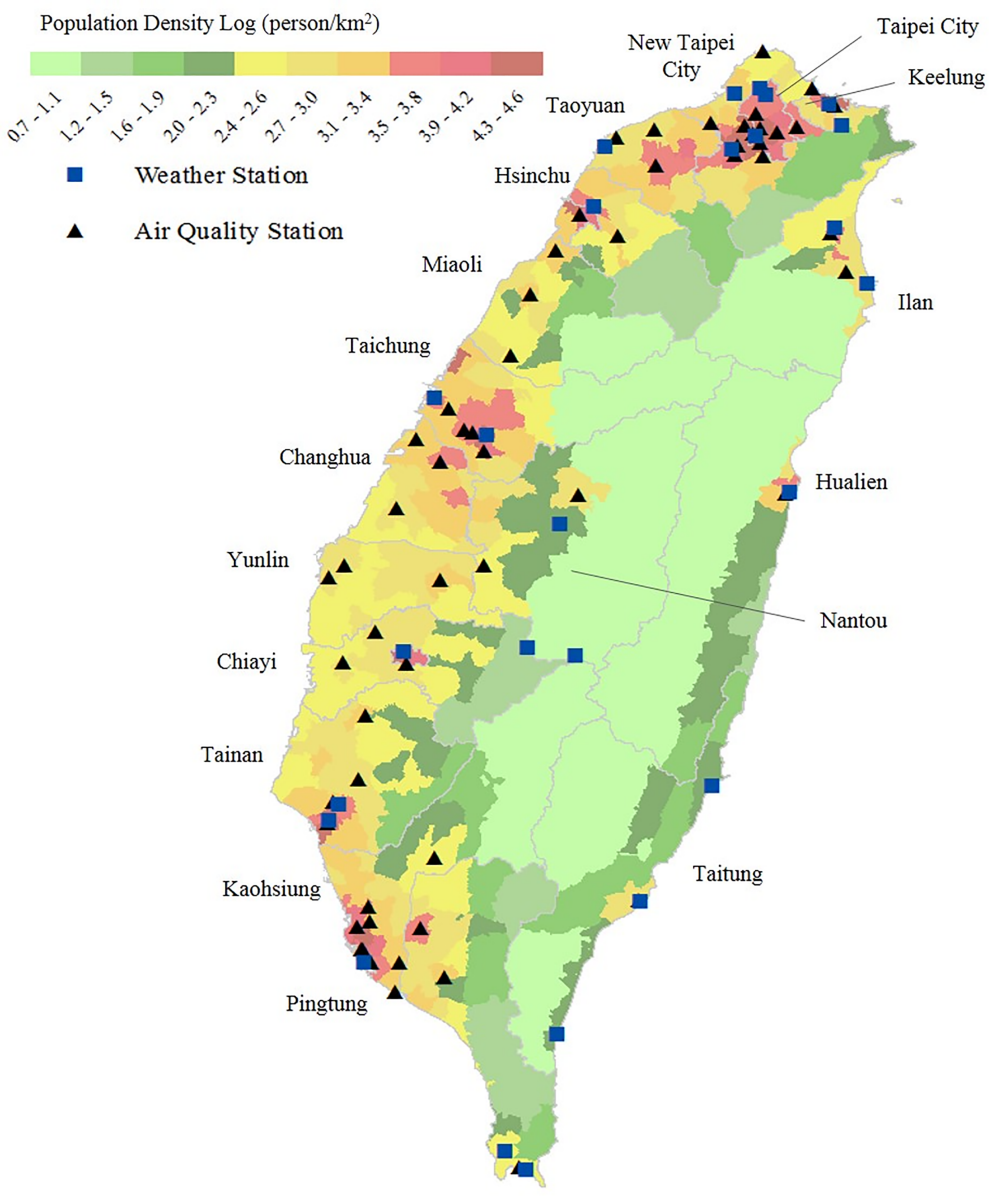
Locations of weather and ambient air quality monitoring stations and population density in Taiwan.

This study used three types of database: health insurance claims of one million people obtained from the National Health Research Institutes (NHRI), daily meteorological records from the Central Weather Bureau, and daily air pollution monitoring records from the Environmental Protection Administration (EPA) for 17 city and county areas island wide from 1996 to 2011. Fig 1 illustrates the population density by district in each area. S1 Table lists information on cardiovascular health status, latitude location and social economics for study areas. In general, population in urban areas (Taipei, New Taipei City, Taichung, and Kaohsiung) had superior wealth and health than those in rural areas (Miaoli, Nantou, Yunlin, Pingtung, Hualien, and Taitung).

The insurance claim database containing medical records of a representative population of one million people randomly sampled from all insured residents for the period of 1996–2012 was provided by the National Health Research Institutes of Taiwan. All identification numbers were scrambled into surrogate number to protect privacy. Disease diagnosed were coded using the ninth revision of the International Classification of Diseases with Clinical Modification (ICD-9 CM). The information on ERVs for OHCA was adopted from previous studies, including consults for paroxysmal ventricular tachycardia (427.1);paroxysmal tachycardia, unspecified (427.2);ventricular fibrillation and flutter (427.4, 427.41, and 427.42);cardiac arrest (427.5);premature beats (427.6);premature beats, unspecified (427.60); supraventricular premature beats (427.61);ventricular premature beats; contractions or systoles (427.69);other specified cardiac dysrhythmias (427.8);sinoatrial node dysfunction (427.81); other specified cardiac dysrhythmias (427.89);cardiac dysrhythmia, unspecified (427.9);sudden death of unknown cause (798);sudden infant death syndrome (798.0);instantaneous death (798.1);death that occurred less than 24 h from symptom onset, not otherwise explained (798.2);unattended death (798.9); and respiratory arrest (799.1) [26, 27].

In addition, to evaluate the role of comorbidities attributable to OHCA risk in association with ambient environment, the historical medical records registered for OHCA cases were retrieved and analyzed. We were particularly concerned with pre-existing heart diseases (391, 402, 404, 415, and 416; 393–398; 420–429; and 785–785.3), ischemic heart diseases (410–414), stroke (430–438), hypertension (401–405) and diabetes mellitus (250).

The Taiwan Central Weather Bureau provided the 24-hour weather information, including average temperature, relative humidity and wind speed, monitored at 25 real-time surface meteorological observatories around Taiwan[28]. Fig 1 displays locations of these weather stations. For area without weather stations, such as Miaoli, Changhua, Yunlin, and Nantou, the weather data were obtained from ambient air quality monitoring stations.

Taiwan EPA established an Air Quality Monitoring Network in 1993, consisting of 74 stationary monitoring stations distributed throughout the island[29]. Concentrations of ambient air pollutants, including PM_10_, PM_2.5_, sulfur dioxides (SO_2_), nitrogen dioxides (NO_2_), ozone (O_3_) and carbon monoxide (CO) and temperature were measured and recorded hourly at each station. The detailed information on the monitoring instruments, stations, and quality assurance criteria is available from the webpage http://taqm.epa.gov.tw/taqm/en/default.aspx. Because PM_2.5_ was measured and recorded island-wide at the beginning of 2005, thus, the risk association was analyzed for 2005–2011. Fig 1 illustrates the locations of the ambient air quality monitoring stations.

### Statistical model

Data analysis was first to count cases of OHCA who had been hospitalized with ERV during 2005–2011. Their characteristics in age and comorbidity were presented. The area-specific associations between the daily ambient environments and ERVs for OHCA were evaluated for the 17 areas using the distributed lag non-linear model (DLNM) with Poisson distribution[30]. The mean ERVs were estimated for 8 groups, including all OHCA cases, OHCA cases aged less than 65 years, OHCA cases aged above 64 years, OHCA cases with pre-existing heart diseases, ischemic heart disease, stroke, hypertension and diabetes mellitus. The group-specific pooled relative risks in association with ambient environments were further estimated by 17 coefficients set for association, i.e. overall cumulative association, lag-specific association, and predictor-specific association, using multivariate meta-analytical second-stage model (restricted maximum likelihood approach) [31].

Sensitivity analyses were performed to evaluate covariates, lag setting, and degrees of freedom (*df*) for single and multi-pollutant models. Akaike’s information criterion was used to select model setting[32]. We found a better model fitting for the single-pollutant model to be adapted in this study. Most previous studies used the single-pollutant model to evaluate the risk of OHCA in exposure to air pollution[10, 13, 15, 16, 18, 23–25]. PM has been linked to the risk of OHCA in many previous studies[10, 15, 16, 22–25]. Therefore, this study mainly displays the risk of OHCA related to ambient daily average temperature and PM concentration. We also evaluated risks associated with extreme daily mean temperatures at the 10^th^ and 95^th^ percentile temperatures (18°C and 30°C, respectively) and at the 1^st^ and 99^th^ percentile temperatures (14°C and 32°C, respectively).

This study evaluated degrees of freedom (*dfs) ranged* from 3 to 20 for the covariates of ambient environment and from 1 to 20 per year for the long-term time smoothing. Analyses showed that the superior models as *dfs* of long-term time trends were set at 7 per year. Daily average temperature and concentrations of the air pollutants PM_10_, PM_2.5_, SO_2_, NO_2_, O_3_, and CO were set at natural cubic spline (NS) with 7 *dfs* to analyze the non-linear effects on the number of ERVs for OHCA. The six-day cumulative effects (lag 0 to lag 5) and the influences on each single day of the air pollutants were then estimated by comparing the concentrations at the standard against the concentrations at the minimum values for daily average of air pollutants_._

Among weather factors, we included relative humidity (RH) and wind speed (WS) in DLNM to control their potential non-linear effects on ERV [33]. Other covariates, including holidays, day of the week (DOW), and daily ERV for pneumonia and influenza (*P&I*, ICD-9 CM: 480–487) were also included in the models.

The relative risks (RRs) and 95% confidence intervals (CIs) of covariates were estimated and reported. We also calculated the daily mean OHCA cases by temperature for the elderly and people < 65 years old and by comorbidity, including diabetes, hypertension, heart disease, ischemic heart disease and stroke. All data manipulation and statistical analyses were performed using SAS version 9.4 (SAS Institute Inc., Cary, NC, USA) and Statistical Environment R (package DLNM ver. 2.2.6 and package mvmeta ver. 0.4.7).

## Results

### Characteristics of ambient environment, social economics, and OHCA

In the cohort of one million population, there were 9,980 cases of OHCA hospitalized with ERV during 2005–2011 (20,568 cases during 1996–2011). Among 9,980 cases, 6,145cases were the elderly and 95.9% cases had comorbidities before hospitalization for OHCA. Among them, 7327 cases had multi-comorbidities and 2,653 cases diagnosed with only one single pre-existing comorbidity. Among cases with one disease, hypertension was most prevalent (n = 1,255), followed by heart diseases (n = 515), diabetes mellitus (n = 408), stroke (n = 253), and ischemic heart diseases (n = 222). Table 1 shows daily ERVs of OHCA by study area and study group from 2005 to 2011. Numbers of ERV of OHCA were higher in urban areas than less urbanized areas, such as the most urbanized metro Taipei (81%) vs. the most rural county Taitung (7%).

**Table 1:** Daily average emergency room visit for out-of-hospital cardiac arrest (standard deviation) in Taiwan, 2005–2011.

	Area Type	All	Elderly (> = 65 years)	Non-elderly (<65 years)	Comorbidity				
					Heart diseases	Ischemic heart diseases	Stroke	Hypertension	Diabetes mellitus
Keelung	Suburban	0.10 (0.32)	0.06 (0.25)	0.04 (0.20)	0.03 (0.19)	0.03 (0.18)	0.02 (0.17)	0.05 (0.22)	0.02 (0.16)
Taipei City	Urban	0.81 (0.90)	0.48 (0.70)	0.32 (0.56)	0.32 (0.60)	0.25 (0.51)	0.19 (0.46)	0.42 (0.68)	0.17 (0.45)
New Taipei City	Urban	0.55 (0.75)	0.30 (0.56)	0.25 (0.50)	0.16 (0.42)	0.13 (0.37)	0.13 (0.37)	0.26 (0.53)	0.11 (0.34)
Taoyuan	Urban	0.40 (0.63)	0.25 (0.50)	0.16 (0.40)	0.13 (0.38)	0.11 (0.36)	0.10 (0.33)	0.22 (0.50)	0.12 (0.36)
Hsinchu	Suburban	0.19 (0.43)	0.11 (0.33)	0.08 (0.29)	0.07 (0.28)	0.05 (0.23)	0.04 (0.21)	0.09 (0.31)	0.03 (0.19)
Miaoli	Rural	0.14 (0.37)	0.09 (0.30)	0.05 (0.22)	0.05 (0.24)	0.04 (0.23)	0.04 (0.24)	0.07 (0.30)	0.03 (0.20)
Taichung	Urban	0.64 (0.82)	0.38 (0.63)	0.26 (0.52)	0.25 (0.53)	0.17 (0.44)	0.15 (0.43)	0.34 (0.62)	0.15 (0.42)
Changhua	Suburban	0.22 (0.47)	0.14 (0.38)	0.08 (0.29)	0.08 (0.33)	0.07 (0.29)	0.05 (0.24)	0.12 (0.37)	0.04 (0.24)
Nantou	Rural	0.12 (0.35)	0.08 (0.28)	0.05 (0.22)	0.04 (0.22)	0.04 (0.20)	0.02 (0.13)	0.06 (0.27)	0.02 (0.16)
Yunlin	Rural	0.12 (0.35)	0.08 (0.28)	0.04 (0.21)	0.04 (0.20)	0.03 (0.17)	0.02 (0.16)	0.06 (0.25)	0.03 (0.18)
Chiayi	Suburban	0.16 (0.42)	0.10 (0.32)	0.06 (0.26)	0.06 (0.25)	0.04 (0.21)	0.04 (0.21)	0.10 (0.30)	0.04 (0.21)
Tainan	Urban	0.32 (0.57)	0.17 (0.41)	0.15 (0.40)	0.10 (0.31)	0.07 (0.27)	0.07 (0.28)	0.15 (0.39)	0.07 (0.28)
Kaohsiung	Urban	0.50 (0.72)	0.27 (0.52)	0.23 (0.50)	0.16 (0.41)	0.13 (0.37)	0.10 (0.33)	0.24 (0.51)	0.11 (0.36)
Pingtung	Rural	0.19 (0.44)	0.10 (0.30)	0.10 (0.31)	0.05 (0.24)	0.04 (0.21)	0.04 (0.22)	0.09 (0.32)	0.04 (0.20)
Ilan	Rural	0.10 (0.32)	0.06 (0.25)	0.04 (0.20)	0.04 (0.22)	0.02 (0.15)	0.02 (0.14)	0.05 (0.24)	0.02 (0.15)
Hualien	Rural	0.14 (0.39)	0.08 (0.30)	0.05 (0.24)	0.05 (0.24)	0.03 (0.19)	0.04 (0.21)	0.09 (0.33)	0.04 (0.23)
Taitung	Rural	0.07 (0.27)	0.04 (0.21)	0.03 (0.16)	0.03 (0.16)	0.02 (0.14)	0.02 (0.13)	0.04 (0.20)	0.02 (0.15)

**Fig 2:**
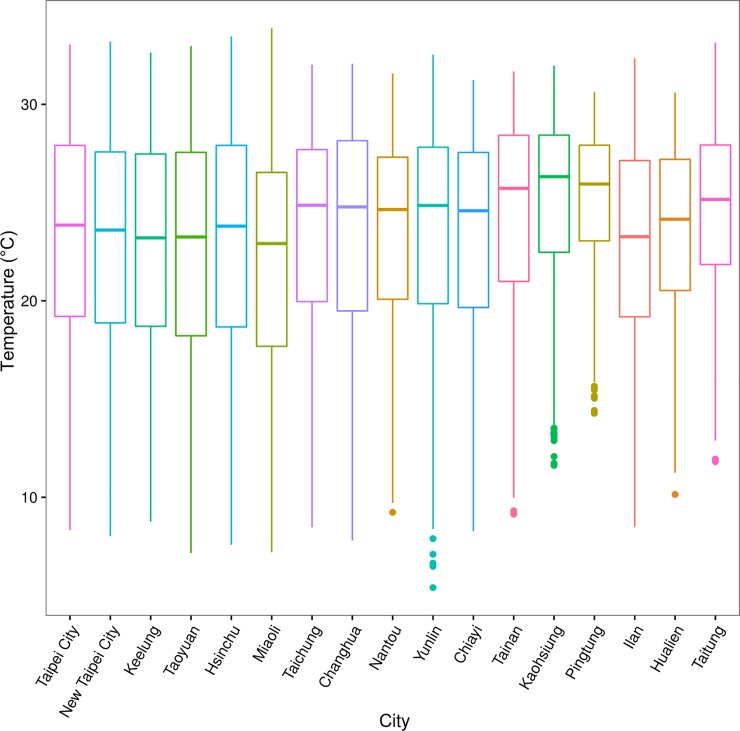
Boxplot of daily average temperature by study area in Taiwan.

Characteristics of the ambient environments are shown in Fig 2 and S1 Fig Average temperature was higher in Southern Taiwan (Tainan, Kaohsiung, and Pingtung), WS was higher in Taoyuan, and RH was similar island wide. S2 Fig shows the concentrations of air pollutants. Mean concentrations of PM_10_, PM_2.5_, SO_2_ and O_3_ were the highest in Kaohsiung, and NO_2_ and CO were the highest in Taipei City.

### Pooled relative risks associated with average temperature

The ambient average temperature showed a stronger association with an elevated risk of OHCA than the 6 air pollutants, which were used to estimate the Pollutant Standards Index. The temperature associated with the lowest risk of OHCA varied among study areas and study subgroups. The present study used 29°C as the reference temperature.

**Fig 3:**
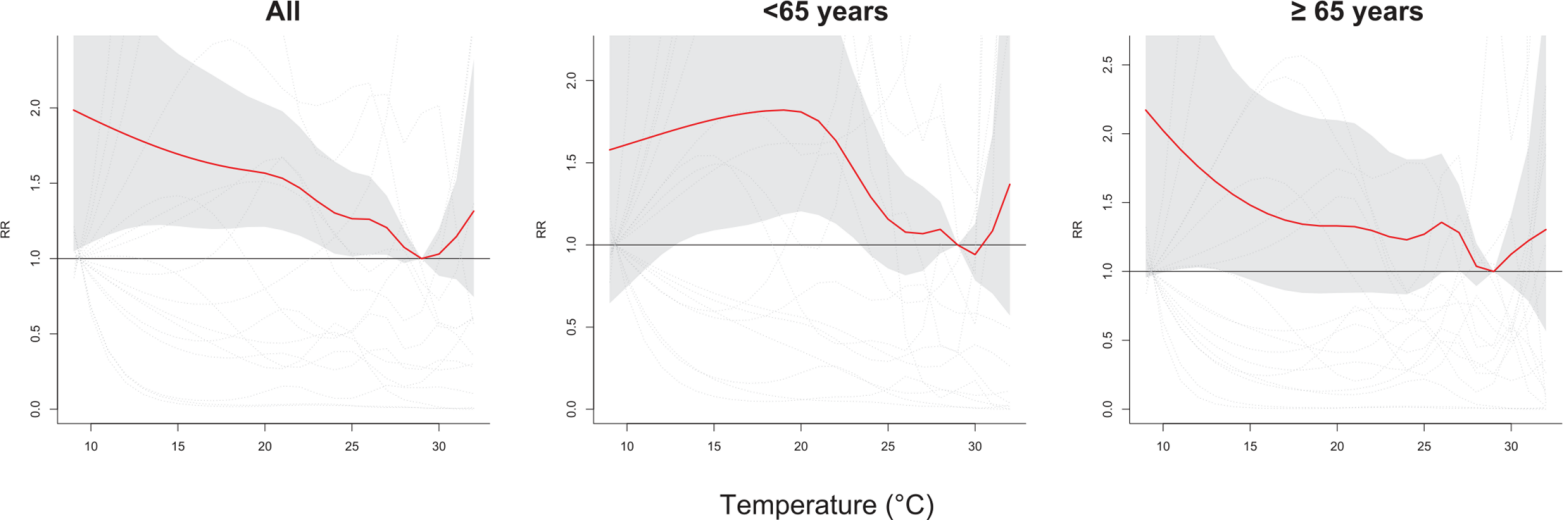
Pooled cumulative 6-day relative risks (RR) of emergency room visit among patients survived with out-of-hospital cardiac arrest in association with daily average temperature for all patients (left), patients younger than 65 years (center), and the elderly (right).

Fig 3 shows the pooled cumulative six-day risk of ERVs of random-effect meta-analyses for all population, population aged less than 65 years, and population aged above 64 years in association with the daily average temperature. Significant risks of OHCA were observed as the temperature was lower than 27°C for whole population, lower than 23°C for population aged less than 65 years, ranged from 11°C to 13°C for the elderly population. The pooled cumulative 6-day ERV RRs were 1.73 (95% CI: 1.22, 2.46) for all OHCA and 1.74 (95% CI: 1.06, 2.84) for population younger than 65 years at temperature of 14°C, and was 1.65 (95% CI: 1.02, 2.69) for the elderly at a 13°C environment.

**Fig 4:**
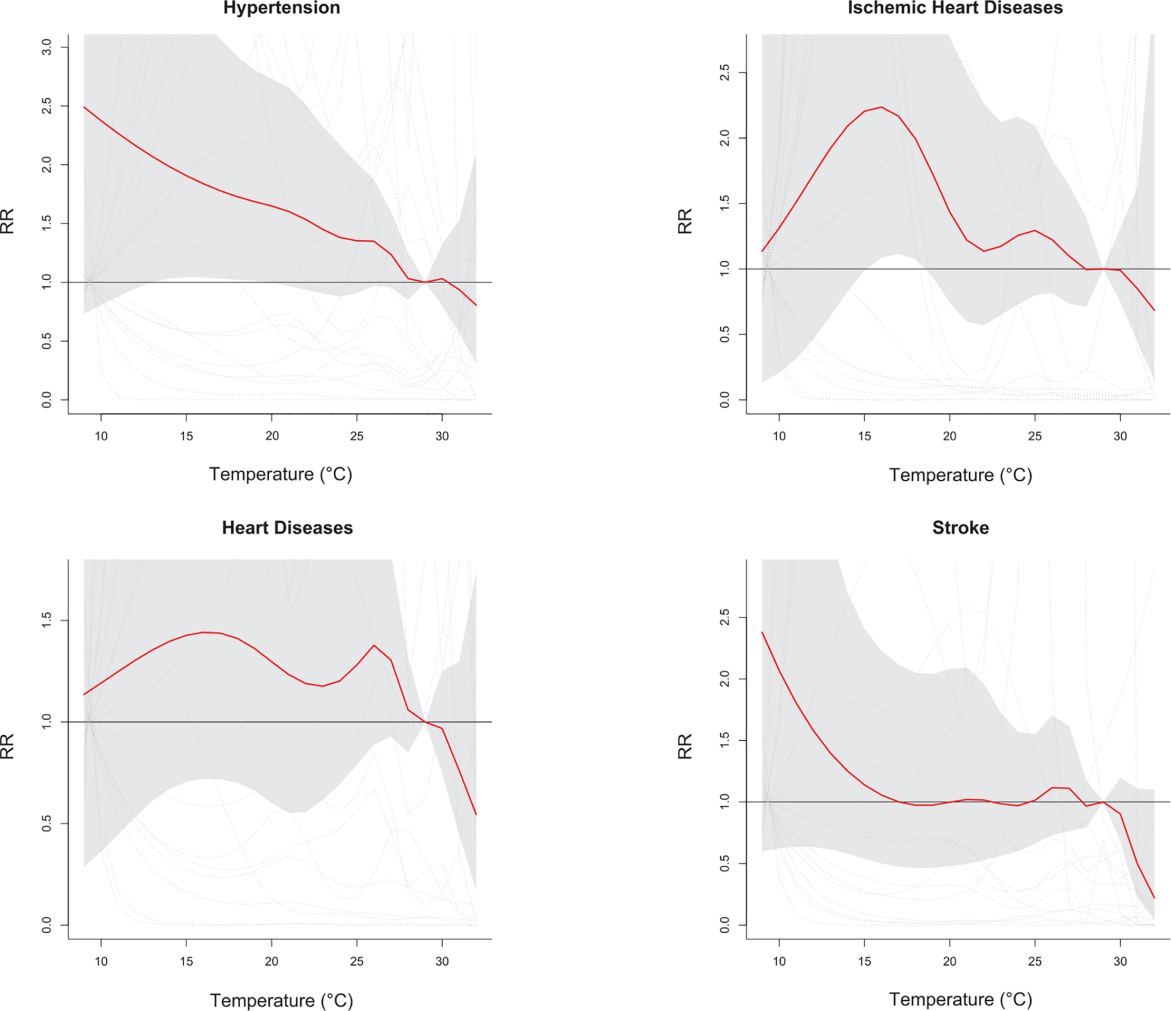
Pooled cumulative 6-day relative risks (RR) of emergency room visit among patients survived with out-of-hospital cardiac arrest in association with daily average temperature for subjects comorbid with hypertension, ischemic heart disease, heart disease, and stroke.

A low temperature (<19°C) significantly associated with an increased number of ERVs for OHCA cases with pre-existing hypertension and ischemic heart diseases, with a cumulative six-day RRs of 1.99 (95% CI: 1.03, 3.87) in a 14°C environment and 2.24 (95% CI: 1.09, 4.60) in a 16°C environment (Fig 4). However, further data analysis showed that low temperature was not significantly associated with the risk for OHCA cases with comorbid heart diseases, stroke and diabetes mellitus.

### Area-specific relative risks associated with ambient environment

Table 2 lists significant ambient environment risk factors in association with ERV of OHCA by study area and group. In general, elevated risks of ERVs for OHCA were more likely associated with daily average temperature, followed by concentrations of PM_10_, PM_2.5_, and NO_2_. The relationship between air pollutants and ERVs for OHCA was stronger in northern Taiwan, including Keelung, Taipei City, New Taipei City, Ilan, and Taoyuan. The ERV risks for OHCA cases with pre-existing hypertension and diabetes mellitus increased with concentrations of air pollutants.

**Table 2:** Ambient environment factors significantly associated with daily emergency room visit for out-of-hospital cardiac arrest in Taiwan.

Study Area\group				Subjects with comorbidity				
	All	> = 65 years	< 65 years	Heart diseases	Ischemic heart diseases	Stroke	Hypertension	Diabetes mellitus
Keelung	Temp/NO_2_	Temp/NO_2_		Temp	Temp	Temp/PM_10_ /O_3_/ NO_2_	PM_10_ /O_3_	PM_10_ /CO
Taipei City	PM_2.5_	Temp	PM_10_ /PM_2.5_	Temp			NO_2_	Temp/NO_2_
New Taipei City	Temp/PM_2.5_	PM_10_ /PM_2.5_	Temp	Temp/PM_10_ /PM_2.5_	PM_10_ /PM_2.5_/CO	Temp/PM_10_ /PM_2.5_	PM_10_	PM_10_ /SO_2_
Taoyuan	O3		O_3_		Temp/O_3_		PM_10_ /O_3_	
Miaoli				Temp/CO				Temp/PM_10_
Taichung						Temp	PM_10_	PM_10_
Changhua					Temp/NO_2_			
Nantou						Temp		
Yunlin			Temp/O_3_					
Chiayi					PM_10_	Temp		
Tainan	Temp	Temp	Temp/NO_2_	Temp	Temp			Temp/NO_2_
Kaohsiung	Temp	Temp	PM_2.5_		Temp	PM_10_		
Ilan	Temp/NO_2_	Temp/PM_2.5_	Temp	Temp/PM_10_ /O_3_	Temp/PM_10_ / NO_2_/CO	Temp/NO_2_	PM_10_ /PM_2.5_/NO_2_	
Hualien	Temp	Temp	Temp	Temp	Temp			O_3_
Taitung			NO_2_	Temp				PM_2.5_/SO_2_

Temperature: Daily average temperature

**Fig 5:**
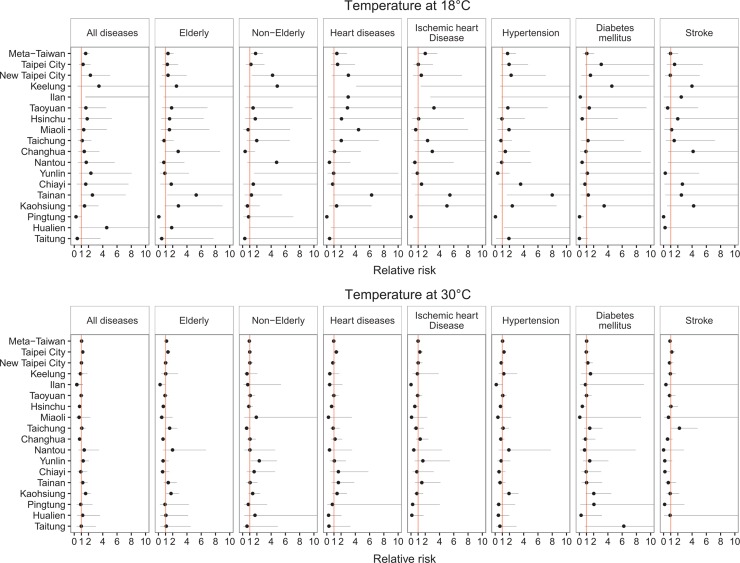
Pooled and area-specific cumulative 6-day relative risks (RR) of emergency room visit among patients survived with out-of-hospital cardiac arrest associated with 18 and 30°C environment.

Pooled area-specific cumulative six-day risks of ERVs for OHCA by study group in association with extreme daily mean temperatures at 18°C and 30°C environments are shown in Fig 5. Significantly elevated ERVs risks appeared at the mean temperature of 18°C in New Taipei City, Keelung, Ilan, Yunlin, Tainan, and Hualien, especially for OHCA cases comorbid with ischemic heart disease and hypertensions. The risk patterns by cold were consistent in lag days, most peak at lag 0 day.

The risk of ERV for OHCA cases associated with high temperature varied with the lag days. Significant risks associated with high temperature were observed in Pingtung (all OHCA cases, cases aged less than 65 years, and cases with pre-existing heart diseases) as lag effects were accumulated for 0 to 3 days. Risks of lag for 0 to 4 or 0 to 5 days were also significant for the elderly with OHCA and cases with comorbid heart disease in Taipei City, for cases with comorbid stroke in Taichung, for population aged less than 65 years in Yunlin, and for all OHCA cases, cases aged 65 years and above, and cases with comorbid hypertension in Kaohsiung.

Pooled and area-specific cumulative six-day risks of ERV for OHCA cases by study group in association with environments of extreme daily mean temperatures at 14°C and 32°C are shown in S3 Fig. Extremely low temperature significantly elevated the ERV risks at the mean temperature of 14°C in Ilan, Miaoli, Tainan, Kaohsiung, and Hualien, especially for OHCA cases comorbid with heart diseases, ischemic heart diseases, and hypertensions. On the contrary, ERV was not associate with extremely high temperature.

**Fig 6:**
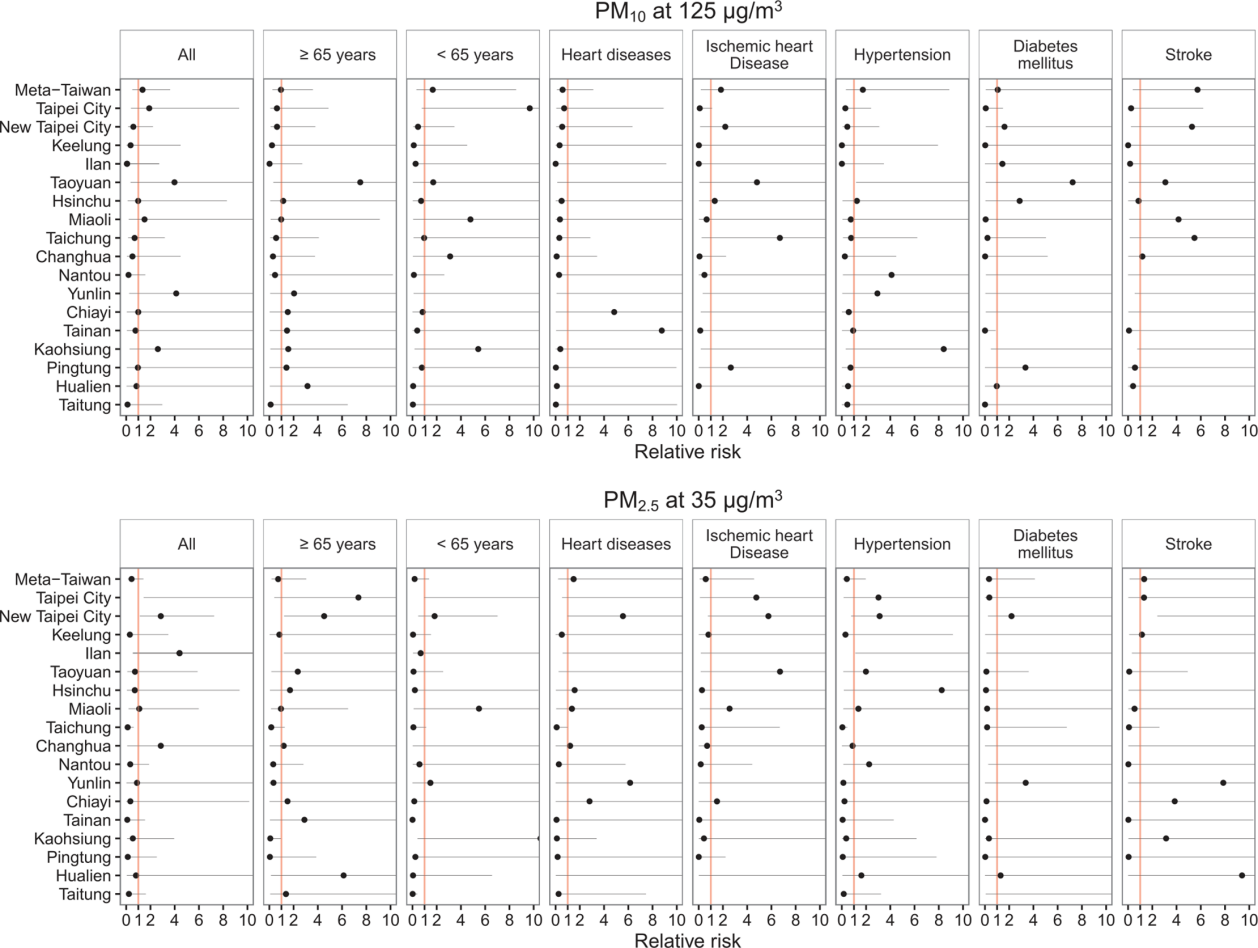
Pooled and area-specific cumulative 6-day relative risks (RR) of emergency room visit among patients survived with out-of-hospital cardiac arrest in association with particulate matter less than 10μm in aerodynamic diameter at 125 μg/m^3^ (reference at 9 μg/m^3^) and particulate matter less than 2.5 μm in aerodynamic diameter at 35 μg/m^3^ (reference at 3μg/m^3^).

Fig 6 displays pooled and area-specific cumulative six-day risks of ERV for OHCA cases by study subgroup in association with PM_10_ at 125 μg/m^3^ (reference at 9μg/m^3^) and PM_2.5_at 35 μg/m^3^ (reference at 3 μg/m^3^). Increased ERV for OHCA significantly associated with PM_2.5_ concentrations in Taipei City (all OHCA), New Taipei City (all OHCA, cases aged 65 years and above, cases comorbid with stroke), and Ilan (cases aged 65 years and above, cases comorbid with hypertension).

**Fig 7:**
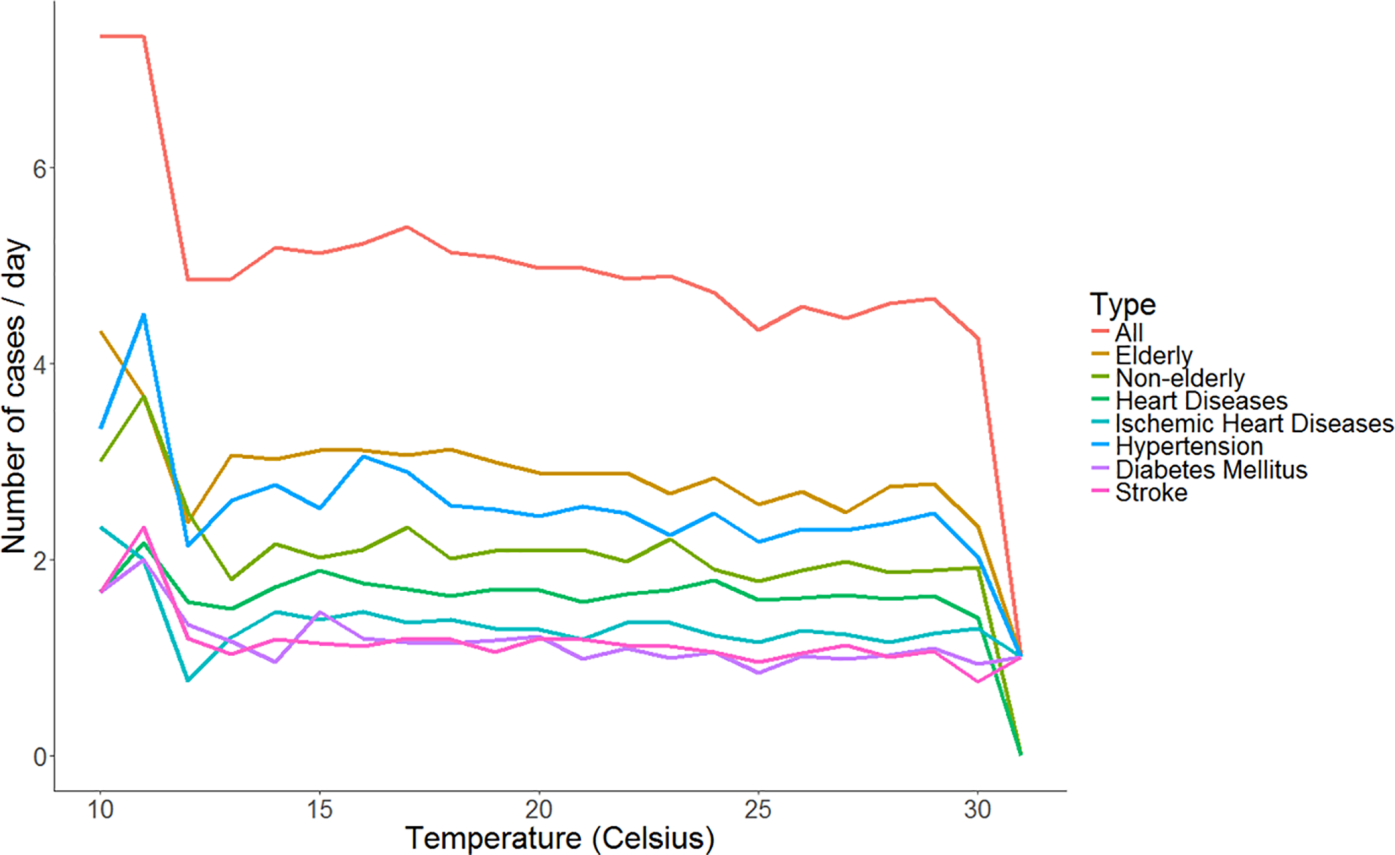
Mean daily OHCA cases reported by temperature for associated groups by age and comorbidity.

Fig 7 shows the mean daily OHCA cases reported by temperature for associated groups. The mean daily cases of OHCA increased sharply in every group when the ambient temperature dropped to 12°C; the overall mean daily cases of OHCA increased to 7.3 cases from 4.8 cases.

## Discussion

Our data revealed that the greatest risk of ERV for OHCA patients was associated with the extreme low temperature in this subtropical island, especially in the rural area, such as Ilan, Miaoli, and Hualien. Among air pollutants, PM_10_ has the greatest association with the risk of OHCA, followed by ambient NO_2_. These relationships were apparent in northern Taiwan, and particular for Taipei City and New Taipei City. Low temperature was significantly associated with increased ERV risk for OHCA cases comorbid with hypertension and ischemic heart disease. On the contrary, ambient air pollutants were significantly associated with ERV risk for OHCA cases who had pre-existing diabetes mellitus, hypertension, and ischemic heart disease. These findings revealed that relationships between ambient environment and ERV risk of OHCA may be modified by regional environmental characteristics and health status of suspected population.

Low temperature may increase blood viscosity, arterial pressure, and plasma cholesterol concentration, thereby intensifying stress on the cardiovascular system[34]. In addition, the effect of heat stress on human body is related to skin blood flow (cardiac output, splanchnic, and renal circulations), sweat rate, and nitric oxide bioavailable in human vasculature[35]. Therefore, exposure to an extreme temperature may elevate the fatal risk for individuals with impaired function of thermoregulation, such as the elderly, and patients of diabetes mellitus, hypertension, and related heart diseases. In Taiwan, the impact is the greatest when the ambient temperature dropped to 12°C; the daily mortality from all causes may increase for 34%.

Cardiovascular diseases are prevalent in patients with OHCA[36]. Previous study reported approximately half of sudden cardiac deaths were unexpected and without pre-existing coronary heart diseases[36][36]. This study showed that 4.10% (409/9980) of OHCA subjects had been hospitalized for OHCA with chronic airway obstruction or pneumonia or bronchitis. This finding deserves further study.

Studies investigating the impact of ambient environmental conditions on OHCA associated with demographics and comorbidity had inconsistent findings[7, 11, 15, 23]. A Stockholm study reported that age and sex had no role in the relationship between OHCA and ambient temperature[7]. The elderly and male population seem to have a higher risk of OHCA in association with ambient temperature in South Korea[11]; comorbidities have a minor role to modify the relationship. However, another Korean study found that individuals with diabetes mellitus and hypertension were vulnerable to high ambient concentration of PM_2.5_ [23][23]. An Italian study proposed that no significant association between ambient PM_10_ concentrations and OHCA cases with malignant neoplasm, diabetes mellitus, anemia, hypertensive diseases, acute myocardial infarction, ischemic heart disease, stroke, diseases of arteries, arterioles and capillaries, pneumonia, and chronic pulmonary diseases[15]. Even though some data revealed the relationships between OHCA and comorbidities, such as diabetes mellitus and heart disease[3–6].The present study demonstrated that the association between ambient temperature and ERV risk appeared mainly for subgroups with hypertension and ischemic heart disease, while ambient air pollutants was associated with OHCA cases with pre-existing diabetes mellitus, hypertension, and ischemic heart disease.

This study assessed the OHCA risk for subgroups with defined comorbidity. Over 73.4% (7,327/9,980) of our study population were comorbid with multiple diseases. Previous studies have indicated that weather related impact is significantly associated with diabetes mellitus, coronary heart disease, and ischemic stroke[3, 37, 38]. To the best of our knowledge, no report has ever evaluated the effects of ambient environment on cases with multiple comorbidities. Future study should evaluate the risk and mechanism for individuals with multiple comorbidities associated with the climate changes and other ambient conditions.

There are some explanations for linking ambient environment with the variation of OHCA risk among study areas. Morbidity, acclimatization and adaptive capacity to extreme temperatures are likely important factors for susceptible population to deal with the impact of climate changes. Prevalence rates for heart diseases, stroke, hypertension, and diabetes mellitus are higher in rural area of Taiwan (S1 Table). In addition, previous studies indicated that socioeconomic status significantly associated with the risk of OHCA[39] and the comorbidity a patient has [40]. The present study showed that population living in area with better economic status, such as in Taipei City (S1 Table), suffer less impacts from ambient temperature than in areas with lower economics in Taiwan.

The risk of ERV for OHCA patients is related with not only local ambient air quality (concentrations and composition), climate, time spent outdoor, but also their physiological characteristics. Previous studies indicated that risk for OHCA in association with concentrations of air pollutants varied among study areas, air pollutants under study, lag structure, and cause of OHCA at a high degree of health heterogeneity[12, 22].

However, we observed that the risk associated with ambient environment in areas with lower socioeconomic statuses, such as Miaoli, Nantou, Yunlin, Pingtung, Ilan, Hualien, and Taitung, were weaker than that in areas with high socioeconomic status, such as Taipei metropolitan. Although the medical service is easily accessed and provided throughout Taiwan, the ERVs for OHCA can be potentially influenced by the locations of hospitals with emergency medical services. S1 Table shows that medical service resources remain less available in rural areas in Taiwan; the risk of ERV of OHCA may not be properly detected in those areas. A previous study in Taiwan indicated the OHCA cases delivered to regional hospitals in non-metropolitan area would have higher mortality[2].

The present study holds several strengths. The 99% coverage rate of the Taiwan National Health Insurance program and widely installed stations for monitoring ambient temperature and air quality ensure a representative study population and reliable environmental data. Confounding effects associating with holidays, week days, long-term trend, and other risk factors associating with pneumonia and influenza had been considered in the models.

This study also has some limitations. First, the information on hospital admission was not individual-based data. Information on patients’ willingness to travel to the hospital, smoking and drinking, and indoor/outdoor ratio of air pollutants that may modify risks associating with ERVs for OHCA patients was were unavailable in the claims data[41–45]. This study has not assessed the risk of OHCA in association with hourly concentrations of air pollutants[13, 16, 20–22, 24, 25] because the ERV data were reported by date instead of hourly. Furthermore, because limited ERV patients were comorbid with pneumonia and influenza (ICD9 CM 480–487; n = 184) and chronic obstructive pulmonary disease and related conditions (ICD9 CM 490–496; n = 374), this study was unable to evaluate the OHCA risk associating with these respiratory disorders. The other limitation was that some patients who had an out-of-hospital cardiac arrest were declared deceased at the scene and did not present to the emergency room. Therefore, findings associated with ERV patients may not reflect all OHCA patients.

There are various risk factors relating to OHCA that may complicate the identification of subjects at a high-risk of ERV. Researchers have suggested that additional efforts to establish a warning system are needed for improving OHCA resuscitation[44]. This study presented various vulnerable subgroups at an elevated OHCA risk in association with extreme temperatures and air pollutants. Our findings could benefit public to comprehend the nebulous conditions and to build public health plans and emerging responses of medical service for OHCA events.

## Conclusions

Our data suggested that ERVs are significantly elevated for all OHCA patients and subjects with pre-existing hypertension and ischemic heart disease associated with the exposure to low temperature in multivariate meta-analytical second-stage model. On the contrary, high temperature is associated with elevated OHCA events in limited subgroups with regional variations. Ambient air pollutants also posed a higher association with ERV for OHCA patients in northern Taiwan. The present study also revealed that populations susceptible for OHCA are more likely at the heterogeneity of risks. Our data provide clinicians support to advice their patients to exercise caution when the weather changes, especially for those who have pre-existing hypertension and diabetes mellitus. It is also important to note that OHCA cases in rural area are less likely to present to the emergency room. Public health authority and emerging medical services also need pay attention to the impact of climate change in health services policy and strategy.

## Supporting information

S1 Fig Boxplots of daily relative humidity (%) and wind speed (m/s) by study area in Taiwan. (TIFF)

S2 Fig Boxplot of daily concentrations of PM_10_, PM_2.5_, SO_2_, NO_2_, O_3_, and CO by study area in Taiwan. (TIFF)

S3 Fig Pooled and area-specific cumulative 6-day relative risks (RR) of emergency room visit of out-of-hospital cardiac arrest in association with 14 and 32°C environment. (TIFF)

S1 Table Information on land, social economics, and medical service for study areas. (DOCX)

## Abbreviations

CI: confidence interval
DLNM: distributed lag non-linear model
ERV: emergency room visits
P&I: pneumonia and influenza
OHCA: out-of-hospital cardiac arrest
PM_10_: particulate matter less than10μm in aerodynamic diameter
PM_2.5_: particulate matter less than 2.5μm in aerodynamic diameter
RR: relative risk
RH: relative humidity
WS: wind speed
