# Supplementary figures and images for: Pre-existing comorbidity modify emergency room visit for out-of-hospital cardiac arrest in association with ambient environments
Source: PLoS One. 2018 Sep 26;13(9):e0204593. doi: 10.1371/journal.pone.0204593 (PMC6157874; doi:10.1371/journal.pone.0204593)

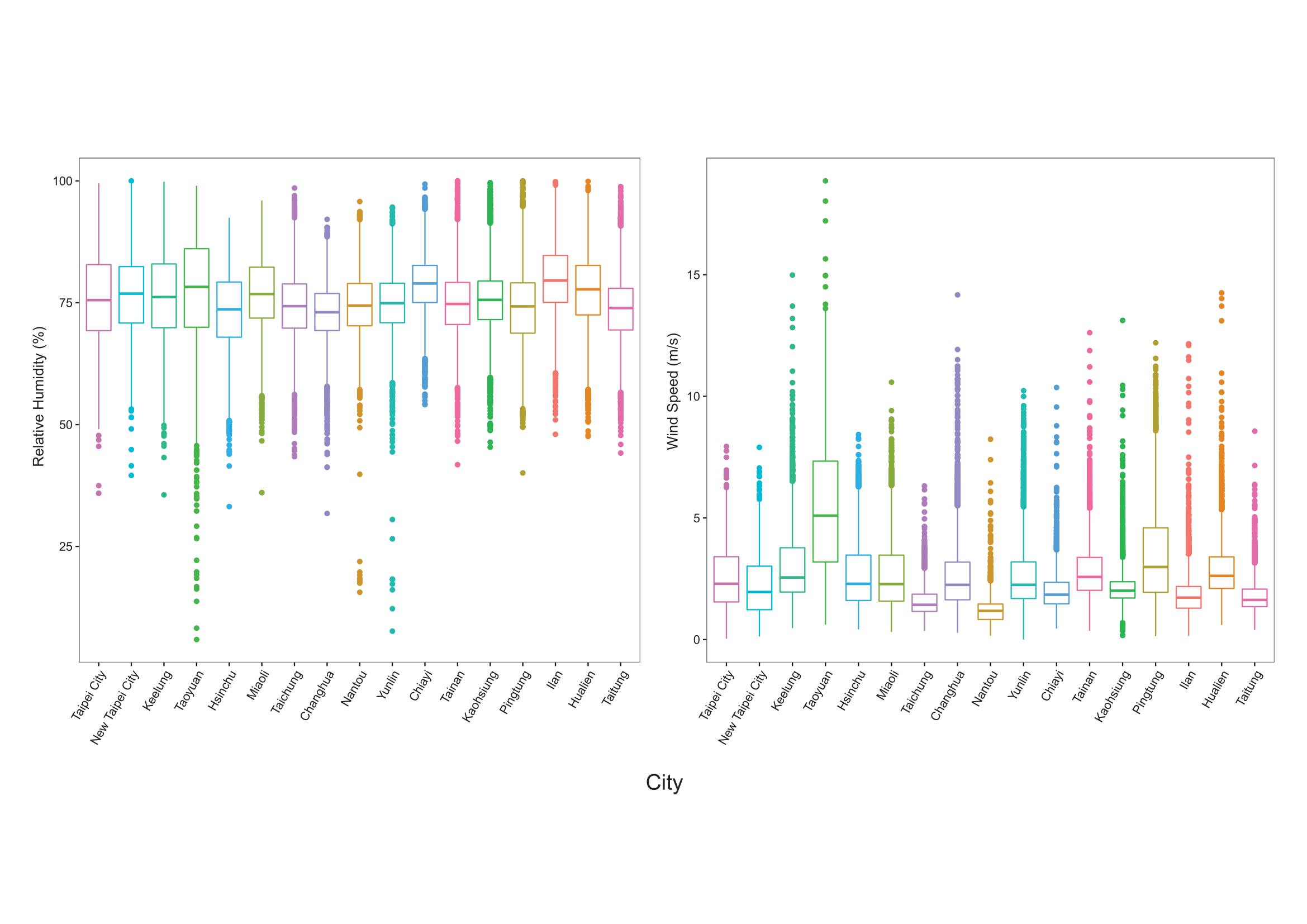

Supplement: S1 Fig — (TIFF) [file pone.0204593.s001.tiff]

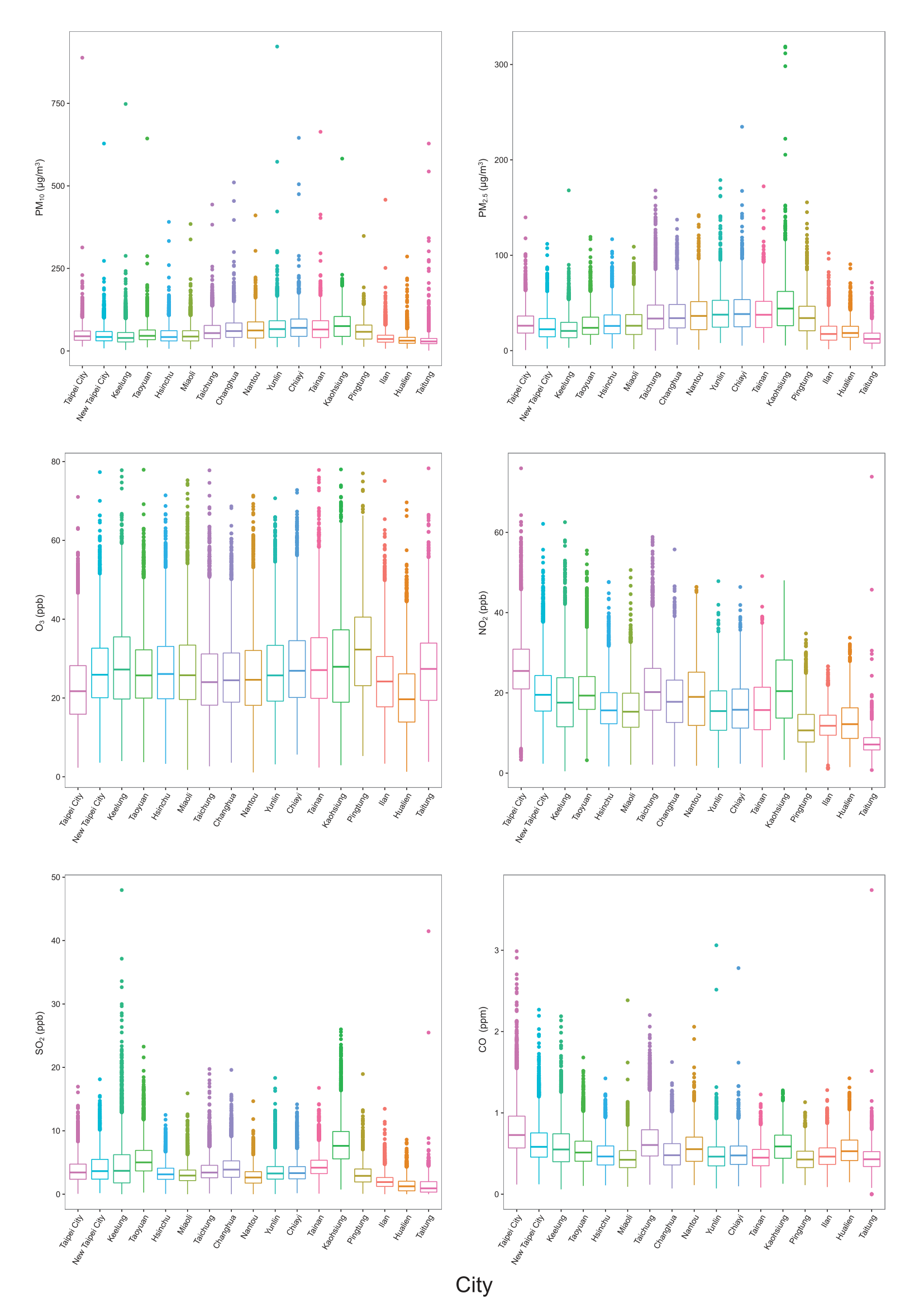

Supplement: S2 Fig — (TIFF) [file pone.0204593.s002.tiff]

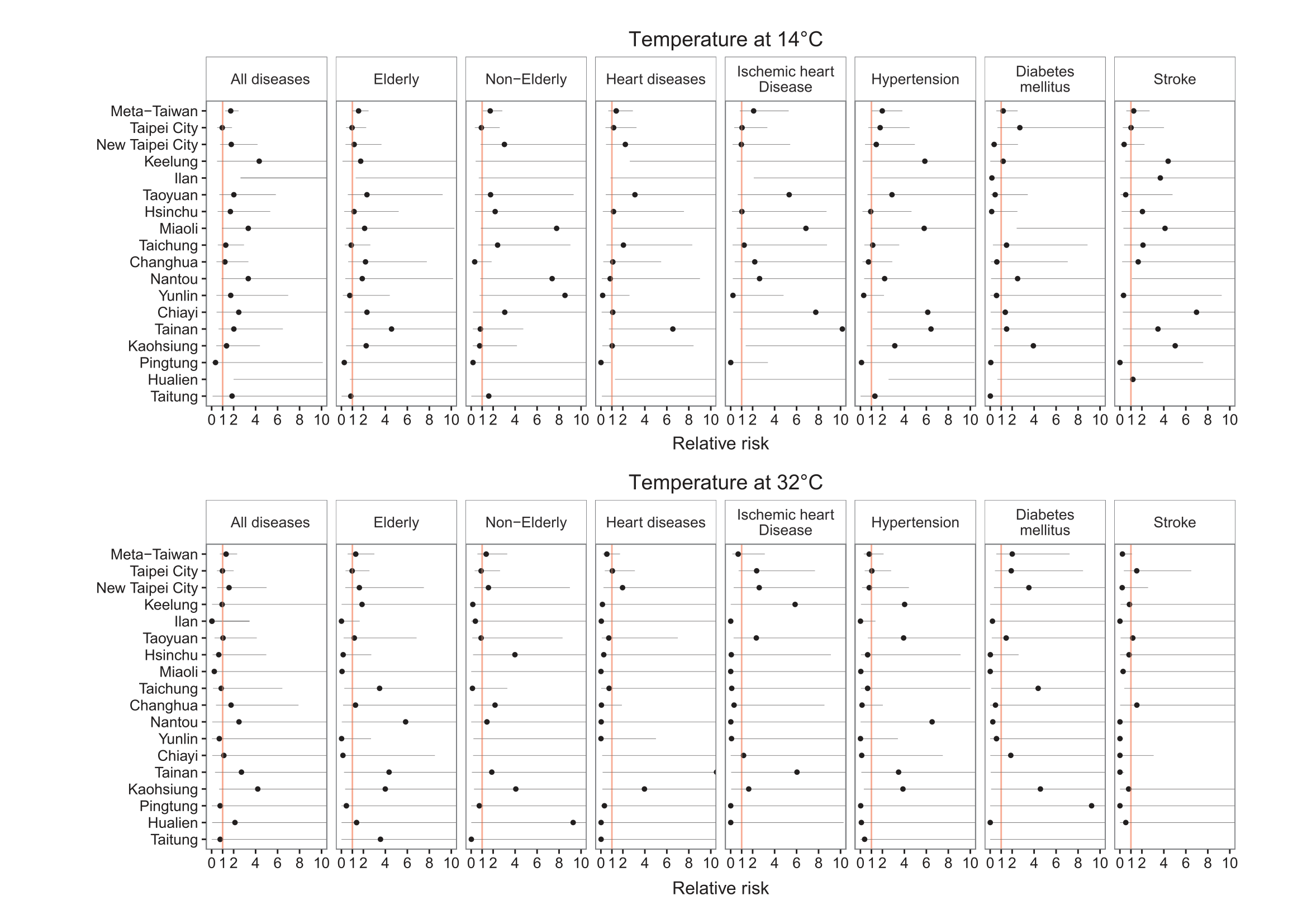

Supplement: S3 Fig — (TIFF) [file pone.0204593.s003.tiff]
