# Supplementary material for: Pre-existing comorbidity modify emergency room visit for out-of-hospital cardiac arrest in association with ambient environments
Source: PLoS One. 2018 Sep 26;13(9):e0204593. doi: 10.1371/journal.pone.0204593 (PMC6157874; doi:10.1371/journal.pone.0204593)
Supplement: S1 Table — (DOCX) [file pone.0204593.s004.docx]

S1 Table. Information on land, social economics, and medical service for study areas

|  | **Keelung** | **Taipei City** | **New Taipei City** | **Taoyuan** | **Hsinchu** | **Miaoli** | **Taichung** | **Changhua** | **Nantou** | **Yunlin** | **Chiayi** | **Tainan** | **Kaohsiung** | **Pingtung** | **Ilan** | **Hualien** | **Taitung** |
| --- | --- | --- | --- | --- | --- | --- | --- | --- | --- | --- | --- | --- | --- | --- | --- | --- | --- |
| Prevalence rate of heart diseases, 2014 (1/100,000) * | 26,906 | 25,184 | 22,504 | 19,219 | 20,557 | 26,658 | 21,002 | 25,203 | 29,239 | 29,205 | 27,666 | 24,178 | 23,544 | 26,224 | 28,928 | 28,095 | 30,404 |
| Prevalence rate of Ischemic heart diseases, 2014 (1/100,000) * | 4,459 | 4,876 | 3,990 | 3,138 | 3,341 | 4,706 | 3,281 | 4,698 | 4,983 | 4,897 | 4,404 | 3,892 | 3,607 | 4,056 | 5,182 | 4,130 | 4,783 |
| Prevalence rate of Stroke, 2014 (1/100,000) * | 2,845 | 2,824 | 2,400 | 2,219 | 2,287 | 3,640 | 2,644 | 3,051 | 3,648 | 4,085 | 3,829 | 2,925 | 2,529 | 3,205 | 3,411 | 3,194 | 3,778 |
| Prevalence rate of Hypertension, 2014 (1/100,000) * | 17,417 | 14,907 | 14,289 | 12,754 | 13,302 | 16,925 | 13,742 | 15,921 | 18,496 | 18,821 | 17,976 | 16,097 | 15,991 | 17,829 | 18,040 | 18,614 | 19,849 |
| Prevalence rate of Diabetes mellitus, 2014 (1/100,000) * | 9,882 | 7,425 | 7,464 | 6,584 | 6,661 | 7,946 | 7,061 | 8,108 | 8,734 | 9,762 | 9,632 | 8,402 | 7,985 | 8,786 | 8,936 | 9,470 | 10,521 |
| **Land, social economics, and medical service in 2013*** | | | | | |  |  |  |  |  |  |  |  |  |  |  |  |
| Latitude (°N) | 25N08' | 25N03' | 25N00' | 24N59' | 24N47' | 24N33' | 24N09' | 24N04' | 23N54' | 23N42' | 23N29' | 23N08' | 22N42' | 22N39' | 24N46' | 23N59' | 22N45' |
| Residents (million) | 0.37 | 2.62 | 3.9 | 2.1 | 0.49 | 0.57 | 2.65 | 1.29 | 0.51 | 0.71 | 0.40 | 1.87 | 2.77 | 0.85 | 0.46 | 0.33 | 0.22 |
| Population density (persons/Km^2^) | 2,824 | 9,884 | 1,927 | 1,674 | 2,243 | 311 | 1,220 | 1,206 | 126 | 548 | 2,395 | 859 | 943 | 307 | 214 | 72 | 64 |
| Population aged 65+ years (%) | 12.0 | 13.5 | 9.48 | 8.90 | 10.5 | 13.7 | 9.39 | 12.8 | 14.3 | 15.8 | 14.1 | 12.2 | 11.4 | 13.5 | 13.5 | 13.2 | 13.7 |
| Average income (USD/person year) | 32,621 | 51,320 | 36,810 | 40,043 | 40,354 | 33,008 | 36,549 | 29,918 | 28,912 | 26,767 | 33,581 | 31,839 | 35,918 | 27,519 | 31,159 | 30,642 | 25,583 |
| Higher education rate for people aged 15 years and above (%) | 36.9 | 69.2 | 39.6 | 36.4 | 42.1 | 27.9 | 41.1 | 29.1 | 28.9 | 25.5 | 38.2 | 36.1 | 40.2 | 28.2 | 30.7 | 31.5 | 20.9 |
| Elderly living alone (%) | 8.34 | 1.38 | 1.09 | 1.35 | 1.06 | 1.51 | 0.95 | 1.34 | 4.47 | 2.07 | 2.01 | 2.2 | 1.45 | 2.07 | 1.12 | 3.38 | 8.04 |
| Air conditioning installation (number/household) | 1.94 | 2.32 | 2.37 | 2.18 | 2.61 | 1.96 | 2.03 | 1.91 | 1.13 | 1.95 | 2.07 | 2.01 | 2.25 | 1.89 | 2.06 | 1.83 | 1.48 |
| No. of Public and Private Hosp. & Clinics | 297 | 3,450 | 3,126 | 1,523 | 390 | 385 | 3,294 | 1,059 | 429 | 503 | 339 | 1,851 | 2,869 | 647 | 338 | 274 | 162 |
| Pop. Served Per Public and Private Hosp. & Clinics (Person) | 1,262 | 1,265 | 795 | 1,363 | 1257 | 1,469 | 820 | 1,224 | 1,206 | 1,407 | 1312 | 1,017 | 969 | 1,317 | 1,356 | 1,219 | 1,388 |
| Area Served Per Public and Private Hosp. & Clinics (Km^2^) | 0.45 | 0.08 | 0.66 | 0.81 | 2.17 | 4.73 | 0.67 | 1.01 | 9.57 | 2.57 | 3.6 | 1.18 | 1.03 | 4.29 | 6.34 | 16.9 | 21.7 |
| Medical Care Personnel Per 10,000 Population (Person) | 101 | 179 | 76.9 | 110 | 97.9 | 76.5 | 126.4 | 98.4 | 86.6 | 84.0 | 154 | 118 | 128 | 98.3 | 110 | 146 | 99.2 |
| No. of Beds in Public and Private Hosp. & Clinics | 2,877 | 24,241 | 16,267 | 14,788 | 2,533 | 3,316 | 20,194 | 7,759 | 3,314 | 3,983 | 3,792 | 12,023 | 20,776 | 6,093 | 4,196 | 4,509 | 1,565 |
| Bed Per 10,000 Population (Beds) | 76.7 | 90.2 | 41.1 | 72.4 | 53.9 | 58.6 | 74.8 | 59.9 | 64.1 | 56.3 | 109 | 63.8 | 74.7 | 71.5 | 91.5 | 135 | 69.61 |

* Data is obtained from The Ministry of Health and Welfare
